# Baroreflex Sensitivity Predicts Response to Metoprolol in Children With Vasovagal Syncope: A Pilot Study

**DOI:** 10.3389/fnins.2019.01329

**Published:** 2019-12-13

**Authors:** Chunyan Tao, Xueying Li, Chaoshu Tang, Hongfang Jin, Junbao Du

**Affiliations:** ^1^Department of Pediatrics, Peking University First Hospital, Beijing, China; ^2^Research Unit of Clinical Diagnosis and Treatment of Pediatric Syncope and Cardiovascular Diseases, Chinese Academy of Medical Sciences, Beijing, China; ^3^Department of Medical Statistics, Peking University First Hospital, Beijing, China; ^4^Department of Physiology and Pathophysiology, Peking University Health Science Center, Beijing, China; ^5^Key Laboratory of Molecular Cardiovascular Sciences, Ministry of Education, Beijing, China

**Keywords:** baroreflex sensitivity, metoprolol, therapeutic response, vasovagal syncope, children

## Abstract

**Objective:** To explore the role of baroreflex sensitivity (BRS) in the head-up tilt test (HUTT) in predicting the therapeutic response of vasovagal syncope (VVS) patients to metoprolol.

**Materials and Methods:** Vasovagal syncope patients treated with metoprolol were enrolled in this study and were classified as responders or non-responders according to changes in their symptom scores before and after metoprolol treatment. Values of BRS in the supine position and at positive response occurrence in the HUTT were obtained, and BRS changes from supine to positive response occurrence were calculated. Differences between responders and non-responders were analyzed. Receiver operating characteristic curve analysis was performed to assess the value of BRS for predicting the therapeutic efficacy of metoprolol in pediatric patients with VVS.

**Results:** Forty patients (14 boys; 11.8 ± 2.5 years) diagnosed with VVS were recruited in the study, 28 of whom were verified to be responders to metoprolol and 12 of whom were verified as non-responders. They did not show any differences in baseline characteristics and hemodynamics in the HUTT (*p* > 0.05). However, the responders had an obviously increased supine BRS value compared to the non-responders (16.9 ± 7.7 ms/mmHg vs. 7.6 ± 3.8 ms/mmHg; *p* < 0.01). No difference in BRS at positive response occurrence was observed between the two groups (8.9 ± 8.5 ms/mmHg vs. 10.6 ± 9.8 ms/mmHg; *p* > 0.05). Accordingly, the changes in the BRS of responders were more obvious than in non-responders (8.0 ± 7.8 ms/mmHg vs. −3.0 ± 10.4 ms/mmHg; *p* < 0.01). The area under the receiver operating characteristic curve for the predictive value of supine BRS was 0.887 (95% CI, 0.779–0.995; *p* < 0.01). A cut-off value of 10 ms/mmHg yielded a sensitivity and specificity of 82 and 83%, respectively, in predicting the therapeutic efficacy of metoprolol in pediatric VVS patients. The area under the receiver operating characteristic curve for the predictive value of BRS changes was 0.827 (95% CI, 0.693–0.962; *p* < 0.01). A cut-off value of 4 ms/mmHg yielded a sensitivity and specificity of 71 and 83%, respectively.

**Conclusion:** Baroreflex sensitivity may predict the response of children with VVS to metoprolol.

## Introduction

Vasovagal syncope (VVS) is common in children and significantly affects their quality of life (Ng et al., 2019). Its underlying mechanisms may involve hypovolemia, autonomic dysfunction, vasomotor dysfunction, baroreceptor reflex abnormalities, serotonin surges, etc. Thus, salt and fluid intake, orthostatic training, and treatment with alpha-agonists, beta-blockers, and selective serotonin reuptake inhibitors are commonly used in clinical practice (Shen et al., 2017; Brignole et al., 2018; Wang et al., 2018). However, unselective treatment cannot ensure a favorable prognosis and indeed might have unexpected adverse effects (Alegria et al., 2003; Sheldon et al., 2006; Romme et al., 2011). Therefore, predicting responsiveness to the therapies before treatment and then selecting the proper medication has become an important topic in this field (Chun et al., 2016; Song et al., 2018; Tao et al., 2019a). Although the mechanisms of VVS are not fully understood, excessive sympathetic tone is considered to be important in some cases, which provides a rationale for the use of beta-blockers as an indispensable therapeutic strategy (Sehra et al., 1999; Alehan et al., 2002; Pitzalis et al., 2003; Raj et al., 2016; Lee et al., 2017; Li et al., 2018; Tao et al., 2019c). There are some pediatric patients with VVS that have no obvious sympathetic activation (Hayoz et al., 1996; Mosqueda-Garcia et al., 1997; Thomson et al., 1997; Flevari et al., 2002; Huang et al., 2017), and beta-blockers only have a class III recommendation in pediatric VVS because of their inconsistent efficacy as well as their adverse effects (Shen et al., 2017; Brignole et al., 2018). The above facts suggest that there be an urgent need to seek out useful indicators to predict the patients who will respond to beta-blocker therapy before treatment.

The baroreceptor reflex is the most important nervous regulatory reflex for maintaining blood-pressure homeostasis, and it plays a pivotal role in blood flow during change between postures. Measurement of baroreflex sensitivity (BRS) could estimate the responsiveness of baroreceptors during an alteration in the arterial pressure and reflect vagal activity to the heart (Dutoit et al., 2010; Okada et al., 2012). BRS represents the cardiovascular autonomic function, and abnormal change in it has been deemed to be a fundamental mechanism for VVS (Lee et al., 2017). Therefore, we hypothesized that BRS might be a useful marker for predicting the therapeutic response to beta-blockers in cases of VVS.

Thus, this study was aimed at assessing the possible role of BRS in the prediction of response to metoprolol, a beta-adrenoceptor blocker, in pediatric patients with VVS.

## Materials and Methods

This retrospective case-control study was performed in the Syncope Unit of Pediatrics at Peking University First Hospital between October 2013 and April 2019. The inclusion criteria were as follows: (1) diagnosed with VVS based on existing guidelines (Wang et al., 2018); (2) having received metoprolol treatment after diagnosis; (3) complete data available on BRS and head-up tilt test (HUTT); and (4) having completed the follow-up process. Children with drug allergies, bronchial asthma, atrioventricular block, or sinus bradycardia were excluded. The particular diagnostic criteria for pediatric VVS were: (1) syncopal attacks, mainly in school-aged children; (2) always induced by predisposing factors, such as prolonged standing, rapid postural changes, stuffy environment, etc.; (3) having a positive response to HUTT; and (4) excluding other diseases that cause syncope (Wang et al., 2018). This study was approved by the Ethics Committee of Peking University First Hospital and was performed in line with the ethical standards expressed in the Declaration of Helsinki. Informed consent was obtained from patients’ parents or guardians.

After neurologic, metabolic, or cardiogenic causes had been excluded, all patients experiencing syncopal episodes took the HUTT between 8:30 AM and 12:00 AM. For the HUTT, a quiet, dimly lit, and warm testing room with resuscitation equipment was prepared in our hospital. Children were asked to fast for at least 4 h in the morning before the test and refrain from any medication that could influence autonomic function for at least 5 times the drug half-life. Children were instructed to lie supine for 10–20 min before being tilted up on an electronic motorized tilt table (HUT-821; Beijing Juchi, Beijing) that had a footboard to obtain stable baseline hemodynamic data. After supine data collection, they were tilted 60° and kept in that position for up to 45 min unless a positive response occurred, which consisted of at least one of the following: (1) obvious hypotension occurring beyond 3 min after tilting (i.e., systolic blood pressure ≤ 80 mmHg, diastolic blood pressure ≤ 50 mmHg, or ≥ 25% decrease in mean blood pressure); (2) bradycardia (i.e., heart rate < 75 bpm in children 4–6 years old, < 65 bpm in those 6–8 years old, and < 60 bpm in those > 8 years old); (3) second-degree or greater atrioventricular block and asystole lasting > 3 s; and (4) sinus arrest (Wang et al., 2018). The hemodynamic patterns of VVS were classified as vasodepressive (notable reduction in blood pressure without an obvious decrease in heart rate), cardioinhibitory (significant heart rate reduction without marked blood pressure decline), and mixed (remarkable decrease in both blood pressure and heart rate) types. No child received venipuncture or administration of medicine (e.g., nitroglycerin and isoproterenol) during the experiment.

Cardiac BRS was calculated by a time-domain sequential method, which was called cross-correlation BRS (xBRS) (Bertinieri et al., 1985; Laude et al., 2004; Westerhof et al., 2004, 2006; Porta et al., 2013b; Chun et al., 2016; Lee et al., 2017). A continuous series of beat-to-beat systolic blood pressure and R-R intervals were monitored by the Finapres Medical System (FMS, FinometerPRO, FMS Co., Netherlands) non-invasively. A finger sphygmomanometer was applied to the left fourth finger of the patient to record blood pressure. Heart rate was counted as the inverse of the interbeat interval. The BeatScope software (Smart Medical, Gloucestershire, United Kingdom) with a mathematical model was used to calculate BRS with the help of hemodynamic data (Li et al., 2018). Ten-second-duration windows of simultaneous systolic blood pressure and interbeat interval series were spline interpolated and resampled at 1 Hz. Each second of time pressure and interval were analyzed for positive cross-correlation and regression slopes using time delays between blood pressure and intervals from 0 to 5 s. The delay with the highest coefficient of correlation was chosen, and the optimal delay (tau) was stored. The slope relating systolic blood pressure with interbeat interval was recorded as an estimate of BRS when the significance of the correlation was < 0.01 (*p* < 0.01). If the conditions were not met, no results were produced for this time duration. Supine BRS was defined as the mean value for 60 s after stable hemodynamics were achieved and positive BRS as the mean value of the positive response duration.

Each enrolled child received metoprolol treatment (0.5 mg/kg/d, twice a day) for a median duration of 3 months. Compliance, reoccurrence, and frequency of syncopal or pre-syncopal episodes and adverse effects during the 3 months after the beginning of treatment were evaluated by the same professionally trained investigator in the outpatient setting or via telephone.

The clinical efficacy of metoprolol was assessed by the changes in symptom scores during the follow-up period of 3 months. The symptom score was based on the occurrence and frequency of syncopal or pre-syncopal episodes: 0, no syncope or pre-syncope; 1, an episode per month; 2, 2–4 episodes per month; 3, 2–7 episodes per week; 4, more than an episode per day. Symptoms were scored for each patient at the beginning of treatment and at 3 months after treatment. A patient was considered a responder if the symptom score was decreased by ≥ 1 and otherwise as a non-responder (Song et al., 2018).

Statistical analysis was performed with SPSS 21.0 (IBM, Armonk, New York). The normality of the distribution of continuous variables was examined by the Shapiro–Wilk test, and they are expressed as mean ± SD and compared by Student’s *t-*test or Mann–Whitney *U*-test between two groups. The difference in BRS between supine and positive response occurrence in HUTT in each group was analyzed by the Wilcoxon Signed-Rank test due to the non-normality of the data. A two-way analysis of variance with repeated measures on ranks was applied to test the change in BRS during HUTT between the two groups. Categorical variables are presented in terms of frequency, and continuity correction was used for comparison. The values of supine BRS and changes in BRS from supine to positive response occurrence in HUTT in predicting the therapeutic effect of metoprolol were evaluated by way of receiver operating characteristic curves, and the cut-off values were determined based on the Youden index. Two-sided *p* < 0.05 was considered statistically significant.

## Results

A total of 40 children (14 boys; 11.8 ± 2.5 years) suffering from VVS with positive responses to HUTT were included in the study in accordance with the inclusion criteria. Among them, 11 patients experienced syncope without specific predisposing factors, while the syncopal episodes of the others were provoked by certain validated factors. Prolonged standing was the most common predisposing factor (19/29), followed by emotional stress (9/29), a stuffy environment (5/29), postural changes (5/29), and others (3/29). Except for three of them, the patients were distressed by typical prodromes before the occurrence of syncope, including but not limited to visual symptoms (21/37), light-headedness (20/37), chest tightness (9/37), diaphoresis (9/37), palpitations (6/37), headache (6/37), nausea (5/37), and fatigue (5/37), for variable durations. Unfortunately, five children suffered from skin abrasion from syncopal attacks, but no other physical injuries were reported.

None of the patients included complained of adverse effects from metoprolol. According to the changes in symptom scores, as mentioned above, 28 children (28/40, 70%) were classified as responders and the other 12 (12/40, 30%) as non-responders. They did not have significant differences in demographics, clinical features, or hemodynamics in HUTT (*p* > 0.05) (Table 1).

**TABLE 1:** Baseline characteristics and hemodynamics in head-up tilt test of responders and non-responders to metoprolol.

		**Non-**		
	**Responders**	**responders**	**χ^2^/*t*/*Z***	
**Items**	**(*n* = 28)**	**(*n* = 12)**	***value***	***p*-Value**
Gender (boys/girls)	11/17	3/9	0.256^†^	>0.05
Age (years)	11.3 ± 2.4	12.8 ± 2.6	1.674	>0.05
Body mass index (kg/m^2^)	19.6 ± 3.8^∗^	19.1 ± 2.6	–0.030	>0.05
Symptom duration before treatment (months)	32.9 ± 33.2^∗^	41.1 ± 39.0	–0.385	>0.05
Symptom scores before treatment (points)	2 ± 1^∗^	2 ± 1^∗^	–0.290	>0.05
Symptom scores at follow-up (points)	0 ± 0^∗^	2 ± 1^∗^	–4.898	<0.01
Treatment duration (months)	3.5 ± 2.1^∗^	3.3 ± 2.0	–0.269	>0.05
Supine HR (bpm)	75 ± 7	79 ± 10	1.343	>0.05
Supine SBP (mmHg)	105 ± 11	106 ± 12	0.232	>0.05
Supine DBP (mmHg)	60 ± 7^∗^	61 ± 6	–0.946	>0.05
Time to positive response in HUTT (min)	22.7 ± 13.5^∗^	29.7 ± 11.7	–1.447	>0.05
HR at positive response occurrence in HUTT (bpm)	99 ± 32	100 ± 34	0.090	>0.05
SBP at positive response occurrence in HUTT (mmHg)	66 ± 13	62 ± 11	–0.893	>0.05
DBP at positive response occurrence in HUTT (mmHg)	43 ± 12	41 ± 13	–0.543	>0.05
Hemodynamic types (vasodepressive/cardioinhibitory + mixed)	21/7	10/2	0.027^†^	>0.05

*DBP, diastolic blood pressure; HR, heart rate; HUTT, head-up tilt test; SBP, systolic blood pressure. Data are mean ± *SD or frequency.*^∗^With non-normal distribution; ^†^Continuity correction.*

**FIGURE 1:**
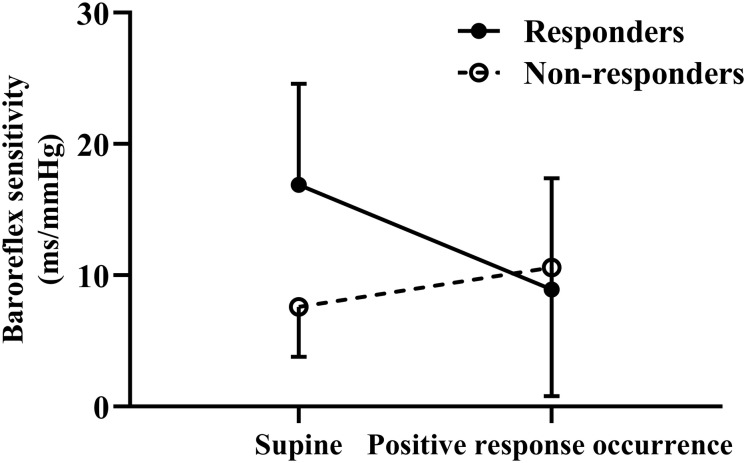
Change in baroreflex sensitivity during head-up tilt test for responders and non-responders to metoprolol.

**FIGURE 2:**
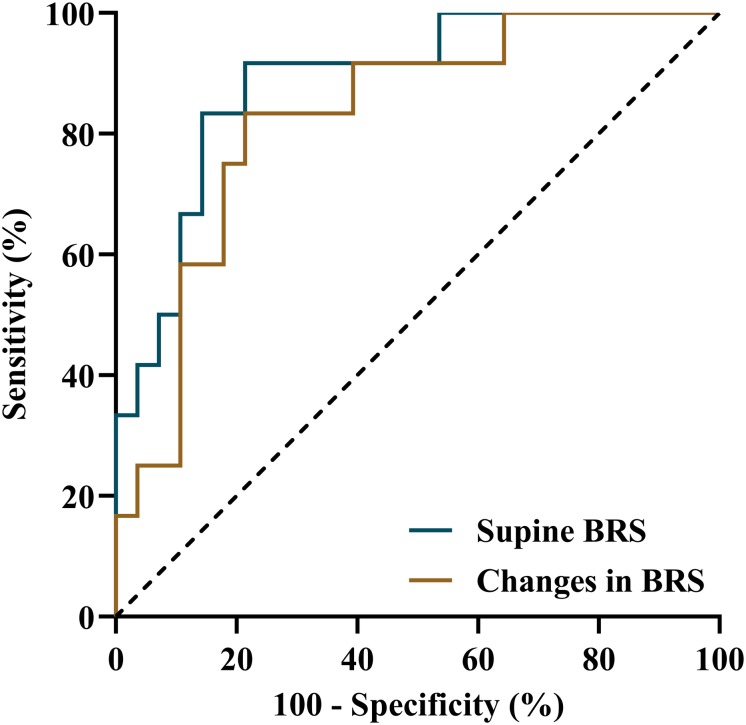
Receiver operating characteristic curves of supine BRS and changes in BRS from supine to positive response occurrence in a head-up tilt test for predicting the therapeutic efficacy of metoprolol for vasovagal syncope in children. BRS, baroreflex sensitivity.

Before treatment, a significantly increased mean supine BRS value was observed in responders compared to non-responders (*p* < 0.01), but their BRS values at positive response occurrence in HUTT did not differ (*p* > 0.05). Accordingly, the changes in BRS from supine to positive response occurrence in HUTT were more obvious in responders than in non-responders (*p* < 0.01) (Table 2). Tested by two-way analysis of variance with repeated measures on ranks, a significant difference in the change of BRS during HUTT was observed between responders and non-responders (*p* < 0.05, Figure 1). Table 2 also shows that, in responders, BRS significantly decreased when positive response occurred (*p* < 0.01), while no significant difference was present in non-responders when positive response appeared (*p* > 0.05). The receiver operating characteristic curve of supine BRS for predicting therapeutic response to metoprolol had an area under the curve of 0.887 (95% CI, 0.779–0.995; *p* < 0.01; Figure 2). The optimal cut-off value was determined as 10.3 ms/mmHg when the Youden index reached the highest. For easy memory, 10 ms/mmHg was eventually chosen, and, in this setting, the sensitivity and specificity were 82% and 83%, respectively. The positive and negative likelihood ratios were 5 and 0.2, respectively (Table 3). Similarly, the receiver operating characteristic curve of changes in BRS for predicting therapeutic response to metoprolol had an area under the curve of 0.827 (95% CI, 0.693–0.962; *p* < 0.01; Figure 2). The highest Youden index was obtained at a cut-off value of 3.7 ms/mmHg, but for easy memory, we determined 4 ms/mmHg as the cut-off value, with a sensitivity and specificity of 71% and 83%, respectively. Accordingly, the positive and negative likelihood ratios were 4 and 0.3, respectively (Table 3).

**TABLE 2:** Comparisons of baroreflex sensitivity in head-up tilt test.

		**Non-**		
	**Responders**	**responders**	***t*/*Z***	
**Items**	**(*n* = 28)**	**(*n* = 12)**	**value**	***p-*Value**
Supine BRS in HUTT (ms/mmHg)	16.9 ± 7.7	7.6 ± 3.8	5.071	<0.01
Positive BRS in HUTT (ms/mmHg)	8.9 ± 8.5^∗^	10.6 ± 9.8^∗^	–0.531	>0.05
Changes in BRS (ms/mmHg)	8.0 ± 7.8	−3.0 ± 10.4	–3.679	<0.01
*Z-*value	−3.803	−0.784	–	–
*p*-Value	<0.01^†^	>0.05^†^	–	–

*BRS, baroreflex sensitivity; HUTT, head-up tilt test; Changes in BRS stands for BRS from supine to positive response occurrence in HUTT. Data are mean ± *SD.*^∗^With non-normal distribution; ^†^Comparison of supine and positive BRS in HUTT in each group.*

**TABLE 3:** The values of supine BRS and changes in BRS in head-up tilt test in predicting the therapeutic response of vasovagal syncope patients to metoprolol.

**Items**	**Cut-off values**	**Clinical response**	
		**Responders**	**Non-responders**
		**(*n* = 28)**	**(*n* = 12)**
Supine BRS (ms/mmHg)	>10	23	2
	≤10	5	10
Changes in BRS (ms/mmHg)	>4	20	2
	≤4	8	10

*BRS, baroreflex sensitivity. Changes in BRS stands for BRS from supine to positive response occurrence in head-up tilt test.*

## Discussion

In this study, we found that the supine BRS of responders was much higher than that of non-responders (*p* < 0.01), and the changes in BRS from supine to positive response occurrence in HUTT were more obvious in responders than in non-responders (*p* < 0.01). Both of these values were able to predict the therapeutic response of VVS patients to metoprolol. However, we recommend the supine BRS as a more suitable indicator due to its ease of performance and relatively higher predictive value.

The putative mechanisms of VVS are heterogeneous, and abnormality in the baroreceptor reflex is considered to be one of the contributing factors. Physiologically, upon standing, increased gravitational forces lead to decreased venous return, cardiac output, and blood pressure, which are detected by baroreceptors in arterial and cardiopulmonary tissues. Signals from these baroreceptors are delivered to the central nervous system, which regulates reflexive cardiovascular activity via changes in sympathetic and parasympathetic outflow with an attempt to maintain normal blood pressure and cerebral perfusion (Mosqueda-Garcia et al., 2000). Some studies showed that the sympathetic nervous system was over-activated in some VVS patients in a supine position (Sehra et al., 1999; Alehan et al., 2002; Tao et al., 2019c). In such a position, excessive sympathetic tone could be compensated for by activating the parasympathetic nervous system to restore a stable hemodynamic condition (Shim et al., 2014). Nonetheless, during standing, physiological adjustment lowers the parasympathetic activity, and the overstimulation of the sympathetic nervous system contracts the ventricular muscle with an “empty chamber.” This vigorous contraction, in turn, triggers an inhibitory response, resulting in hypotension and/or bradycardia and, eventually, reduced cerebral blood flow, leading to the occurrence of VVS (Nair et al., 2003; Salo et al., 2007; He et al., 2018).

Beta-adrenoceptor blockers can reduce the sympathetic tone, then decreasing cardiac contractility and increasing the ventricular volume to cure VVS (Dendi and Goldstein, 2002; Benditt et al., 2003). However, not all patients have an overactive cardiovascular sympathetic nervous function. In our study, the supine BRS of non-responders was significantly lower than that of responders, and almost no changes were observed when positive response occurred, showing a similar change trend to healthy volunteers (Lee et al., 2017). Under such a condition, patients do not have excessive sympathetic activation, and the use of metoprolol would have no benefits. It could even be harmful because of its role in decreasing sympathetic activity and increasing peripheral venous volume and cardiac preload (Verheyden et al., 2008; Vallurupalli and Das, 2010; Vyas et al., 2013). Therefore, unselective use of beta-adrenoceptor blockers in VVS patients is not suggested (Tao et al., 2019b; Xu and Wang, 2019). Gielerak and colleagues aimed to predict the efficacy of continuous propranolol (a beta-blocker) by observing tilt test results after intravenous administration of propranolol. The outcomes were more favorable for patients with than without a negative response or delayed positive response in a subsequent tilt test (Gielerak et al., 2005). However, the method used in the study by Gielerak et al. is complex in clinical practice. In 2019, Kong et al. attempted to predict therapeutic response to metoprolol in VVS children with 24-h urine norepinephrine and found that patients with 24-h urine norepinephrine > 34.84 μg/24 h seemed to have a better prognosis (Kong et al., 2019). However, this approach was not time-effective. In the present study, we demonstrated that BRS had the ability to predict VVS patients’ response to metoprolol and, more importantly, that it is non-invasive and easy to perform in a clinical setting.

Detection of BRS is not only beneficial to patients who would potentially receive beta-blocker treatment but also to those receiving orthostatic training. Orthostatic training is considered to cure patients with VVS accompanied by sympathetic nervous dysfunction, another subset of patients with autonomic nervous activity alterations (Tan et al., 2010; Mitro et al., 2018). Both Tan et al. and Mitro et al. found that BRS was increased after the intervention of orthostatic training, and furthermore, Mitro et al. identified that VVS patients with lower upright BRS had better outcomes than those with relatively higher values. Meanwhile, other studies demonstrated that VVS patients had different BRS statuses (increased, decreased, or unchanged BRS compared to that of healthy controls) (Thomson et al., 1997; Flevari et al., 2002; Pitzalis et al., 2003; Lee et al., 2017; Li et al., 2018). As such, all patients are suggested to undergo BRS examination to understand their autonomic nervous function and allow the formulation of individualized treatment strategies.

Associations have been identified between BRS and adverse outcomes under some pathological conditions, such as coronary artery disease, heart failure, hypertension, etc. (Ranucci et al., 2017; Bari et al., 2019). Varied BRS impacts some VVS patients, but whether it is a risk factor for relevant diseases, like hypotension, hypertension, heart attacks, etc., in the long term follow-up has not been determined.

Baroreflex sensitivity quantifies the magnitude of the reflexive R-R interval response to systolic blood pressure change only if an effective heart rate response really occurs. In reality, progressive systolic blood pressure changes are not always coupled with progressive reflexive R-R interval modulation in healthy individuals (Di Rienzao et al., 2001). Thus, BRS is not an impeccable index for demonstrating baroreflex control. The baroreflex effectiveness index is a new index capable of quantifying the ratio between the number of systolic blood pressure ramps followed by reflexive R-R interval ramps and the total number of systolic blood pressure ramps (with or without corresponding reflexive R-R interval ramps). It can provide complementary information on baroreflex regulation. Besides, causality analysis between heart rate and blood pressure can provide a more comprehensive description of cardiovascular modulation than BRS (Porta et al., 2013b). Whether the baroreflex effectiveness index and causal relations between heart rate and blood pressure play valuable roles in the management of VVS is worthy of study. xBRS, a time-domain sequential method, was proposed by Westerhof et al. (2004, 2006). It was applied in the present study because of its advantages of allowing a much larger number of estimates to be obtained more regularly over time and having half the estimation variance of some other methods, such as the sequential BRS method, as tested on the EUROBAVAR database (Westerhof et al., 2004). In addition, the BRS measured by this approach is highly correlated to BRS values calculated by other approaches, such as spectral techniques using low-frequency or high-frequency bands and the trigonometric regressive spectral analysis method, and it is capable of detecting low BRS values in patients disturbed by cardiac baroreflex failure (Laude et al., 2004). Actually, the measurement of BRS can be performed in different ways, evoked or spontaneous, sequential or spectral. The cross-correlation method used in the study is not the sole method that can be exploited for BRS assessment from spontaneous variations, and is not able to discriminate between 10-s-rhythm bands and oscillations in a ventilator (Porta et al., 2013a). Truly universal agreement on the way of evaluating BRS bias and precision is still needed.

The present study has some limitations. Firstly, recall bias could not be completely avoided because of the study design. Secondly, the sample size was not large enough in view of statistics, and the characteristics of the population may limit the generalizability to other populations. Therefore, this pilot study limited the power to some extent of the conclusion. Besides, a lack of BRS during blood pressure and/or heart rate re-stabilization after positive response in HUTT limited the full comprehension of the autonomic nervous activity changes of the VVS patients. In addition to using the BRS that we detected in the study to reflect the baroreflex control, there are other indexes such as the baroreflex effectiveness index and causal relations between heart rate and blood pressure that were not used in the present study due to the unavailable equipment. However, our results, for the first time, showed that VVS children with a supine BRS of > 10 ms/mmHg or a change in BRS from supine to positive response occurrence in HUTT of > 4 ms/mmHg were suggested to have a favorable response to metoprolol. Larger-scale multicenter investigations are required to validate the predictive value of BRS in the prognosis of VVS patients after intervention with metoprolol. We hope that the findings will be useful for implementing individualized therapy in pediatric VVS.

## Data Availability Statement

The datasets generated for this study are available on request to the corresponding author.

## Ethics Statement

This study was approved by the Ethics Committee of Peking University First Hospital and performed in line with the ethical standards expressed in the Declaration of Helsinki. Informed consent was obtained from patients’ parents or guardians.

## Author Contributions

CYT had primary responsibility for the protocol development, patient enrollment, data collection, preliminary data analysis, and writing the manuscript. XL designed the data collection progress, revised the data analysis, and reviewed and revised the manuscript. CST gave important advice on study design, supervised data collection, and reviewed the manuscript for important intellectual content. HJ and JD supervised the design and execution of the study, checked the data analysis, contributed to the writing of the manuscript and had final approval on the manuscript submitted. All authors read and approved the final manuscript and agreed to be accountable for all aspects of the work. No prior presentation of the study data has been made in posters or abstracts.

## Conflict of Interest

The authors declare that the research was conducted in the absence of any commercial or financial relationships that could be construed as a potential conflict of interest.

## Abbreviations

BRS: baroreflex sensitivity
HUTT: head-up tilt test
VVS: vasovagal syncope
